# ﻿Diversity of *Cladosporium* (Cladosporiales, Cladosporiaceae) species in marine environments and report on five new species

**DOI:** 10.3897/mycokeys.98.101918

**Published:** 2023-06-02

**Authors:** Wonjun Lee, Ji Seon Kim, Chang Wan Seo, Jun Won Lee, Sung Hyun Kim, Yoonhee Cho, Young Woon Lim

**Affiliations:** 1 School of Biological Sciences and Institute of Microbiology, Seoul National University, Seoul 08826, Republic of Korea Seoul National University Seoul Republic of Korea

**Keywords:** deep sea, Dothideomycetes, intertidal zone, marine fungi, open sea, Republic of Korea, taxonomy, Western Pacific

## Abstract

*Cladosporium* species are cosmopolitan fungi, characterized by olivaceous or dark colonies with coronate conidiogenous loci and conidial hila with a central convex dome surrounded by a raised periclinal rim. *Cladosporium* species have also been discovered in marine environments. Although many studies have been performed on the application of marine originated *Cladosporium* species, taxonomic studies on these species are scarce. We isolated *Cladosporium* species from three under-studied habitats (sediment, seawater, and seaweed) in two districts including an intertidal zone in the Republic of Korea and the open sea in the Western Pacific Ocean. Based on multigenetic marker analyses (for the internal transcribed spacer, actin, and translation elongation factor 1), we identified fourteen species, of which five were found to represent new species. These five species were *C.lagenariiforme***sp. nov.**, *C.maltirimosum***sp. nov.**, *C.marinum***sp. nov.** in the *C.cladosporioides* species complex, *C.snafimbriatum***sp. nov.** in the *C.herbarum* species complex, and *C.marinisedimentum***sp. nov.** in the *C.sphaerospermum* species complex. Morphological characteristics of the new species and aspects of differences with the already known species are described herein together with molecular data.

## ﻿Introduction

*Cladosporium* Link is a cosmopolitan genus in the order Cladosporiales of Dothideomycetes (Abdollahzadeh et al. 2020). It is characterized by coronate conidiogenous loci and, conidial hila formed by a central convex dome surrounded by a raised periclinal rim (David 1997). With these characteristics, sequence-based identification and classification have been officially proposed as standardized methods (Seifert 2009; Hibbett et al. 2016). Although the internal transcribed spacer (ITS) region has been selected as the primary fungal barcode marker (Schoch et al. 2012), it has low resolution for *Cladosporium* (Schubert et al. 2007). Therefore, for species-level identification of *Cladosporium*, actin (*act*) and translation elongation factor 1 (*tef1*) genes have been proposed (Schubert et al. 2007; Bensch et al. 2012). Currently, *Cladosporiumsensu stricto* (*s. str.*), the type species of which is *C.herbarum* (Pers.) Link, comprises three species complexes, namely *Cladosporiumcladosporioides*, *Cladosporiumherbarum*, and *Cladosporiumsphaerospermum* species complexes (Schubert et al. 2007; Zalar et al. 2007; Bensch et al. 2010). More than 230 species have been identified (Iturrieta-González et al. 2021), and new species have continuously been proposed based on morphological features and molecular sequence data (Iturrieta-González et al. 2021; Pereira et al. 2022).

Members of *Cladosporium* are commonly found in terrestrial environments, such as caves (Pereira et al. 2022), indoors (Bensch et al. 2018), and soil (Iturrieta-González et al. 2021). They occupy various ecological niches as saprotrophs (Iturrieta-González et al. 2021), endophytes (Hamayun et al. 2009), phytopathogens (Marin-Felix et al. 2017), animal pathogens (Sandoval-Denis et al. 2015), and hyperparasites (Anderson et al. 2016). *Cladosporium* has also been reported in marine environments, such as seawater (Zambelli et al. 2015), sediment (Luo et al. 2020; Marchese et al. 2021), and marine organisms (Godinho et al. 2019; Marchese et al. 2021). Several *Cladosporium* species with halotolerant and osmotolerant characteristics have adapted to marine environments (Zalar et al. 2007; Biango-Daniels and Hodge 2018; Araújo et al. 2020). Marine *Cladosporium* species produce diverse secondary metabolites (Salvatore et al. 2021). Metabolites are being actively studied for potential application in the bioremediation of pollutants, such as polycyclic aromatic hydrocarbons (Birolli et al. 2018) and polyester polyurethane (Zhang et al. 2022). However, such research is conducted on a limited number of species because *Cladosporium* has been poorly investigated in marine environments. Active taxonomic studies on marine *Cladosporium* species are necessary to understand the true diversity of marine *Cladosporium* and to discover valuable metabolites.

As part of exploring marine fungi for the Marine Fungal Resource Bank, we have been continuously isolating marine fungi. In this study, *Cladosporium* strains isolated from various marine habitats were tentatively identified based on morphological characteristics and ITS sequence analysis. The present study was aimed at (1) identifying *Cladosporium* strains at the species level using multigenetic marker analyses (ITS, *act*, and *tef1*) and (2) examining the niche specificity of *Cladosporium* species by district and habitat. Fourteen *Cladosporium* species were identified, of which five were confirmed as novel species – three in the *C.cladosporioides* species complex, and one each in the *C.herbarum* and *C.sphaerospermum* species complexes. A detailed morphological description of the new species along with genetic marker sequences, is presented, herein.

## ﻿Materials and methods

### ﻿Sampling and isolation

Sampling was performed in two separate districts – an intertidal zone of the Republic of Korea and the open sea in the Western Pacific (Table 1). From the intertidal zone of the Republic of Korea, sediments (sea sand and mudflat) were sampled from six sites in 2016–2021, and seaweeds were sampled in two regions in 2021 (Table 1). From the Western Pacific, the top layer of the deep-sea sediment was sampled using two Giant Piston Corers (GPC) and a Multiple Corer. Seawater was sampled in 2021 from three different regions at three depths – the surface layer, the oxygen minimum zone, and the subsurface chlorophyll maximum layer – using Niskin bottles equipped in the conductivity, temperature, and depth (CTD) rosettes (Table 1).

**Table 1.** Information on the sampling sites.

Region	Habitat	District	GPS coordinates
CJ (ChuJa-do)	Seaweed	Republic of Korea	33°58′14″N ~ 33°55′46″N, 126°16′55″W ~ 126°20′31″W
GH (GangHwa)	Sediment (Sea sand)	Republic of Korea	37°35′18″N, 126°26′33″W
	Sediment (Mudflat)	Republic of Korea	37°36′36″N, 126°31′13″W
GS (GoSeong)	Sediment (Sea sand)	Republic of Korea	38°28′39″N, 128°26′22″W
JJ (JeJu-do)	Seaweed	Republic of Korea	33°23′53″N, 126°14′24″W
	Sediment (Sea sand)	Republic of Korea	33°14′21″N, 126°20′02″W
MA (MuAn)	Sediment (Mudflat)	Republic of Korea	35°01′40″N, 126°25′11″W
	Sediment (Sea sand)	Republic of Korea	35°03′44″N, 126°20′13″W
SC (SunCheon)	Sediment (Mudflat)	Republic of Korea	34°50′48″N, 127°29′33″W
	Sediment (Sea sand)	Republic of Korea	34°50′29″N, 127°29′09″W
US (UlSan)	Sediment (Sea sand)	Republic of Korea	35°23′00″N, 129°20′46″W
C1 (CTD1)	Sea water	Western Pacific	17°19′00″N, 149°51′18″W
C2 (CTD2)	Sea water	Western Pacific	16°01′39″N, 151°49′19″W
C3 (CTD3)	Sea water	Western Pacific	19°31′18″N, 151°27′19″W
G1 (GPC1)	Sediment (5730 m)	Western Pacific	15°22′42″N, 151°40′50″W
G2 (GPC2)	Sediment (5730 m)	Western Pacific	20°45′57″N, 152°35′32″W
MC (Multiple corer)	Sediment (5814 m)	Western Pacific	16°06′15″N, 152°25′00″W

Seawater samples in Niskin bottles were filtered immediately through a sterile polycarbonate track-etched (PCTE) membrane filter (d = 47 mm, ϕ = 0.2 μm, GVS Filter Technology, Sanford, USA) using a vacuum pump. The filters were then placed on dichloran rose bengal chloramphenicol (DRBC; Difco) agar, supplemented with sterilized seawater (SSW), to isolate fungi. Seaweed samples were cut into 0.5 × 0.5 cm^2^ pieces and placed on DRBC agar. For the sediment samples, 5 g of each sample was first diluted with 45 mL of SSW in a 50 mL falcon tube, and 5 mL of the mixture was further diluted with 45 mL of SSW. Approximately 150 μL of the dilution was spread on potato dextrose agar (PDA; Difco), DRBC agar, and glucose yeast extract agar (GYA; 1 g glucose, 0.1 g yeast extract, 0.5 g peptone, and 15 g/L). Sabouraud dextrose agar (SDA; Difco) medium was used for the diluted samples from the intertidal zone and open sea. Whenever colonies grew on a medium, each single colony was transferred onto PDA supplemented with SSW to obtain a pure culture. Fungal isolates were stored in 20% glycerol with SSW at −80 °C and deposited in the Seoul National University Fungus Collection (SFC), Seoul, Republic of Korea.

### ﻿DNA extraction,PCR amplification, and sequencing

The fungal mycelium mats of each isolate grown on PDA were ground using a Bead Ruptor Elite Homogenizer (OMNI International, Kennesaw, GA, USA). Genomic DNA was extracted using an AccuPrep Genomic DNA Extraction Kit (Bioneer Co., Daejeon, Korea), following a modified manufacturer’s protocol, in which CTAB Extraction Solution (Biosesang, Korea) was used instead of the TL buffer in the kit.

The ITS region and partial regions of two protein-coding genes, *act* and *tef1*, were amplified using polymerase chain reaction (PCR). Each region was amplified using the following primer sets: ITS1F (Gardes and Bruns 1993) / ITS4 (White et al. 1990) for the ITS region, ACT-512F/ACT-783R (Carbone and Kohn 1999) for *act*, and EF1-728F/EF1-986R (Carbone and Kohn 1999) or EF1-728F/EF2 (O’Donnell et al. 1998; Carbone and Kohn 1999) for *tef1*. PCR was performed using a Maxime PCR premix (iNtRON Biotechnology, Inc., Korea) or an AccuPower PCR premix (Bioneer Co., Korea) on a C1000 Touch Thermal Cycler (Bio-Rad) or a Mastercycler Nexus Gradient (Eppendorf) under the following conditions: initial denaturation at 95 °C for 5 min, 35 cycles of denaturation at 95 °C for 40 s, annealing at 55 °C for 40 s, and extension at 72 °C for 60 s, followed by a final extension at 72 °C for 5 min. The amplicons were validated using gel electrophoresis on a 1% agarose gel. The PCR products were purified using a PCR Purification Kit (GeneAll Biotechnology, Seoul, South Korea) or an ExoSAP-IT Express PCR Product Cleanup (Thermo Fisher Scientific), following the manufacturer’s instructions.

Sanger sequencing was performed in both forward and reverse directions using an ABI Prism 3730xl Genetic Analyzer (Life Technologies, Gaithersburg, MD, USA) by Macrogen (Seoul, Republic of Korea). The generated sequences were proofread and merged using the “*De novo assemble*” function in the Geneious Prime software ver. 2022. 0. 2. (Biomatters Ltd., San Diego, CA, USA). All the assembled sequences were deposited in GenBank (Suppl. material 1: table S1).

### ﻿Phylogenetic analyses

Reference sequences of the genus *Cladosporium* were retrieved from GenBank (Suppl. material 1: table S2). *Cercosporabeticola* (CBS 116456) was used as an outgroup. Sequences were aligned individually for each region using MAFFT v7.450 (Katoh et al. 2019), and manually trimmed at the end. The alignments for ITS, *act*, and *tef1* were then concatenated for downstream phylogenetic analyses. For phylogenetic analyses, ModelTest-NG (Darriba et al. 2020) was used for model selection of each partition. The selected model for ITS was TrNef+I+G4 and that for *act* and *tef1* was TPM2uf+I+G4. As both the models were unsupported in MrBayes, each was replaced with SYM+I+G and GTR+I+G, respectively, according to Dellicour et al. (2014). Maximum likelihood (ML) and Bayesian inference (BI) analyses were performed to generate the phylogenetic trees. The ML tree was inferred using RAxML (Stamatakis 2014) with 1,000 replications and the GTR+G+I model was used after finding the model test in MEGA 7 (Kumar et al. 2016). MrBayes (Ronquist et al. 2011) was used for the BI analysis. BI trees were sampled every 1000^th^ from 10,000,000 generations with four Markov chains. ModelTest-NG and MrBayes were performed on the CIPRES gateway supplied by XSEDE (Miller et al. 2012). The ML phylogenetic tree was selected for data visualization. Posterior probability (PP) values are represented on the ML phylogenetic tree.

### ﻿Morphological observations

Morphological observations of the new species were performed as described by Schubert et al. (2007). The new species were cultured on PDA, malt extract agar (MEA; Oxoid), oatmeal agar (OA; Difco), and synthetic nutrient-poor agar (SNA; KH_2_PO_4_ 1 g, KNO_3_ 1 g, MgSO_4_7H_2_O 5 g, KCl 0.5 g, glucose 0.2 g, saccharose 0.2 g, and Bacto agar 20 g/L) for 14 d at 25 °C in the dark for description of cultural characteristics. The diameters of the colonies were measured using ImageJ (Schneider et al. 2012). The color of the surface and reverse side of the colonies was described using the Methuen Handbook of Colour (Kornerup and Wanscher 1978). For observing microscopic features, such as conidial structures, the representative strains were cultured on SNA at 25 °C in the dark from 7 d to 14 d. Observations were made using a Nikon 80i compound light microscope (Nikon, Tokyo, Japan), and at least 30 measurements were collected per strain using ImageJ (Schneider et al. 2012) at 400× magnification.

## ﻿Results

A total of 88 fungal strains isolated from marine environments were initially identified based on ITS sequences and morphological characteristics as members of the genus *Cladosporium*. A total of 77 strains were from the intertidal zones in the Republic of Korea and 11 were from the open sea in the Western Pacific. For identification at species level, concatenated phylogenetic analysis was carried out using ITS, *act*, and *tef1* sequences. Sequences from 55 of the 88 strains were analyzed together with sequences from 168 reference strains. For phylogenetic analyses, 666 sequences were used – 165 sequences (ITS: 55, *act*: 55, *tef1*: 55) that were newly generated in this study and 501 reference sequences (ITS: 168, *act*: 166, *tef1*: 167). The final combined alignment comprised 1,205 characters, including gaps, of which ITS, *act*, and *tef1* contained 507, 250, and 448, respectively. In the alignment data, 450 conserved (ITS: 332, *act*: 79, *tef1*: 49), 602 variable (ITS: 158, *act*: 155, *tef1*: 289), and 449 informative (ITS: 79, *act*: 128, *tef1*: 242) characters were included.

**Figure 1.**
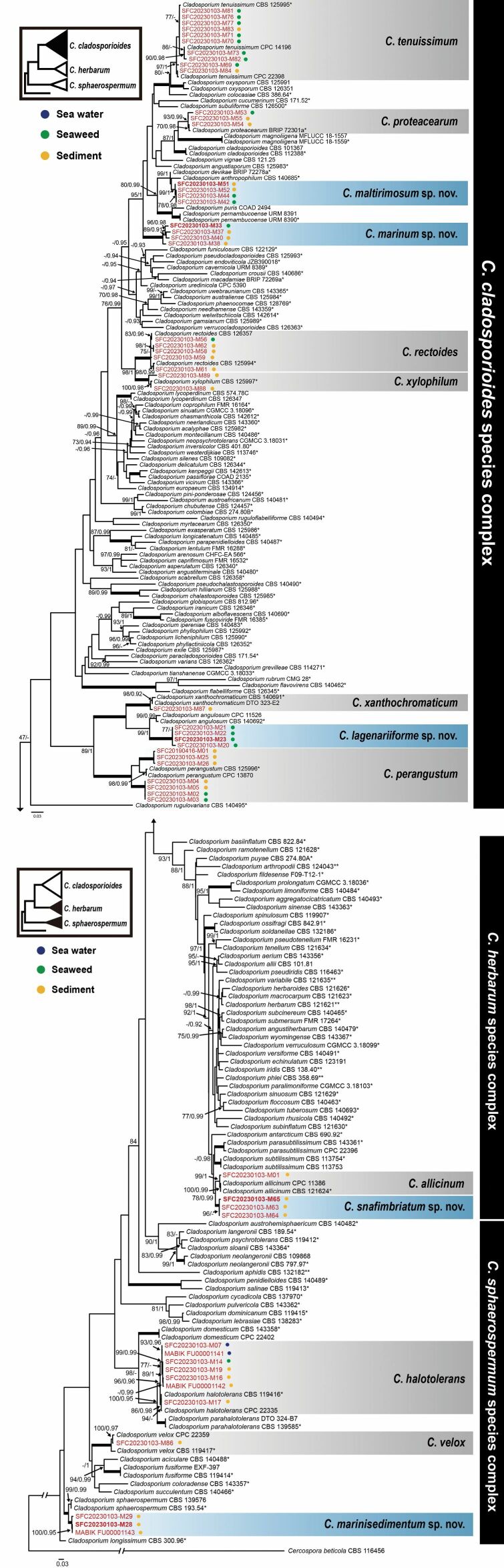
Maximum likelihood (ML) tree of *Cladosporium*, inferred based on ITS, *act*, and *tef1* sequences. The values at each node indicate the bootstrap of ML (≥ 70) and the posterior probability (PP; ≥ 0.90) of Bayesian inference (BI) analyses. Thick lines on branches indicate 100 bootstrap and 1.00 PP support. The tree is rooted to *Cercosporabeticola* (CBS 116456). The small tree on the upper left represents the topology of species complexes of the genus. The filled triangles correspond to the species complex(es) shown. The colored circles symbolize habitats where the species were isolated. Previously described species are grouped in gray boxes, and blue boxes indicate new species. Strains with newly generated sequences are in red, with the holotypes in bold. The holotype and epitype for the reference strains are represented with “*” and “**”, respectively.

The analyzed *Cladosporium* strains belonged to fourteen monophyletic subclades. Nine clades were respectively matched with *C.allicinum*, *C.halotolerans*, *C.perangustum*, *C.proteacearum*, *C.rectoides*, *C.tenuissimum*, *C.velox*, *C.xanthochromaticum*, and *C.xylophilum* described previously (Zalar et al. 2007; Bensch et al. 2010, 2012; Sandoval-Denis et al. 2016; Prasannath et al. 2021), supported by ≥ 93% bootstrap and ≥ 0.96 PP values. The other five taxa were not grouped with any reference sequences and established each distinct clade with ≥ 99% bootstrap and 1.00 PP values (Fig. 1). Therefore, we propose them as new *Cladosporium* species based on their morphological characteristics. Three taxa belong to the *C.cladosporioides* species complex, one to the *C.herbarum* species complex, and one to the *C.sphaerospermum* species complex.

### ﻿Taxonomy

#### ﻿ Cladosporium lagenariiforme

Taxon classificationFungiCladosporialesCladosporiaceae

Wonjun Lee & Y.W. Lim sp. nov.

3CCE8290-D99D-5970-9559-DFB80153311E

 847096

Figs 2A , 3A

##### Typification.

Republic of Korea. Jeju-do, Chuja-myeon, 33°57′11.88″N, 126°18′07.56″E, seaweed, 31 Aug 2021, M.S. Park & Y.W. Lim (holotype SFC20230103-M23, stored in a metabolically inactive state).

**Figure 2.**
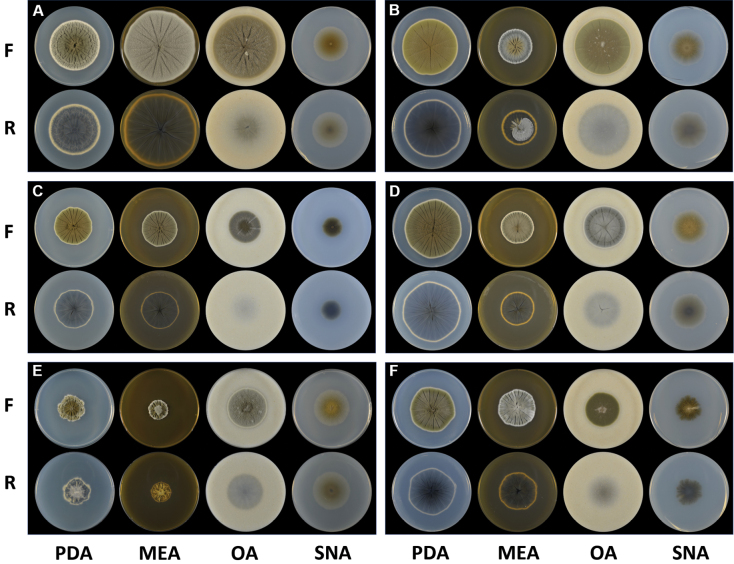
Morphological characteristics of the new and unrecorded species on PDA, MEA, OA, and SNA media **A***Cladosporiumlagenariiforme* sp. nov. **B***Cladosporiummaltirimosum* sp. nov. **C***Cladosporiummarinisedimentum* sp. nov. **D***Cladosporiummarinum* sp. nov. **E***Cladosporiumproteacearum***F***Cladosporiumsnafimbriatum* sp. nov. “F” and “R” represent the front and the reverse sides of the culture plate, respectively. PDA: potato dextrose agar; MEA: malt extract agar; OA: oatmeal agar; SNA: synthetic nutrient-poor agar.

##### Etymology.

The term ‘*lagenariiforme*’ was derived from the generic name of a calabash (*Lagenaria*) to describe the shape of the ramoconidia and secondary ramoconidia.

**Figure 3.**
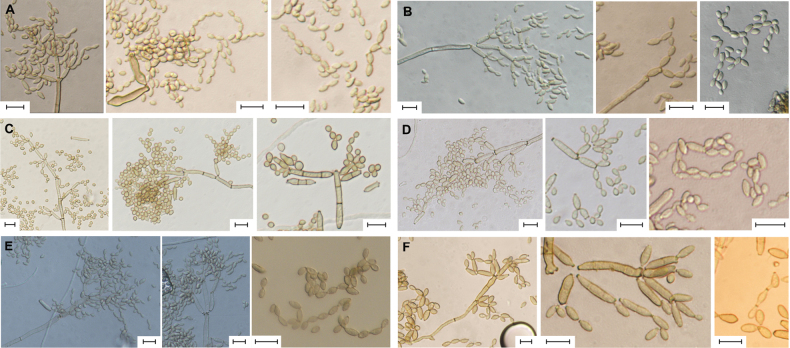
Morphological characteristics of conidial structures on SNA**A***Cladosporiumlagenariiforme* sp. nov. **B***Cladosporiummaltirimosum* sp. nov. **C***Cladosporiummarinisedimentum* sp. nov. **D***Cladosporiummarinum* sp. nov. **E***Cladosporiumproteacearum***F***Cladosporiumsnafimbriatum* sp. nov. Scale bars: 10 μm (**A–F**).

##### Description.

**Asexual morphology: *Mycelium*** mainly immersed, composed of septate, branched, hyaline, verruculose hyphae, 1.8–4.3 μm wide. ***Conidiophores*** macronematous and micronematous, arising laterally from hyphae, sometimes reduced to conidiogenous cells, septate, erect to slightly flexuous, slightly nodulose, branched, up to 208 μm long, 1.7–3 μm wide, pale brown, verrucose. ***Conidiogenous cells*** integrated, terminal and intercalary, cylindrical to subcylindrical, 15.1–37.6 × 1.4–2.9 μm, bearing up to four slightly darkened and refractive conidiogenous loci. ***Ramoconidia*** 0(–2)-septate, subcylindrical to ellipsoidal, obclavate, sometimes calabash-like constricted at the center, 8.7–26.4 × 1.9–3.5 μm [av. (± SD) 15.2 (± 5.46) × 2.6 (± 0.39)], pale brown, verruculose. ***Conidia*** forming branched chains, with up to six conidia in the terminal unbranched part, long neck between conidia, aseptate, pale brown, smooth to verruculose, with protuberant, slightly darkened, and refractive hila. ***Small terminal conidia*** aseptate, ellipsoidal, 2.1–3.5 × 1.3–2.3 μm [av. (± SD) 3 (± 0.32) × 1.9 (± 0.22)]. ***Intercalaryconidia*** aseptate, ellipsoidal to limoniform, 2.9–5.3 × 1.6–2.7 μm [av. (± SD) 4.0 (± 0.47) × 2.0 (± 0.25)]. ***Secondary ramoconidia*** 0(−1)-septate, subcylindrical to ellipsoidal, obclavate, sometimes calabash-like constricted at the center, 7.3–21.6 μm long × 2.0–3.7 μm [av. (± SD) 13.6 (± 4.01) × 2.6 (± 0.36)].

***Cultural characters***: Colonies on PDA 52–70 mm diam after 14 d at 25 °C, greenish gray (1B2) to olive (1E3), reverse dark gray (1F8), floccose, velvety, crateriform, radially furrowed, wrinkled, usually significantly wrinkled in the margin part; margin yellowish gray (4B2) edge, slightly lobate; aerial mycelia abundantly formed, dense, with few exudates, sporulation profuse. Colonies on MEA 46–74 mm diam after 14 d at 25 °C, gray (1C1) to olive gray (1D3), reverse dark gray (1F8), floccose-woolly, umbonate, (frequently) radially furrowed, wrinkled, sometimes significantly wrinkled in the margin part; margin whitish edge, slightly lobate, rarely undulate; aerial mycelia abundantly formed, dense, with prominent exudates, sporulation profuse. Colonies on OA 56–68 mm diam after 14 d at 25 °C, olive yellow (3C8), reverse concolorous, woolly-floccose, raised, radially furrowed; margin yellowish gray (3C2), regular; aerial mycelia abundantly formed, without prominent exudates, sporulation moderate. Colonies on SNA 36–37 mm diam after 14 d at 25 °C, olive yellow (3E8) to olive (3E8), reverse concolorous, powdery, flat; margin yellowish white (2A2), regular; aerial mycelia abundantly formed, without prominent exudates, sporulation profuse.

##### Habitat and distribution.

Isolated from seaweeds; Southern Korean seaside in Republic of Korea.

##### Additional cultures examined.

Republic of Korea. Jeju-do, Chuja-myeon, 33°23′53″N, 126°14′24″E, seaweed, 31 Aug 2021, M.S. Park & Y.W. Lim (SFC20230103-M20, stored in a metabolically inactive state); Jeju-do, Chuja-myeon, 33°23′53″N, 126°14′24″E, seaweed, 31 Aug 2021, M.S. Park & Y.W. Lim (SFC20230103-M21, stored in a metabolically inactive state); Jeju-do, Chuja-myeon, 33°23′53″N, 126°14′24″E, seaweed, 31 Aug 2021, M.S. Park & Y.W. Lim (SFC20230103-M22, stored in a metabolically inactive state).

##### Notes.

*Cladosporiumlagenariiforme* sp. nov. is phylogenetically closely related to *C.angulosum* and *C.xanthochromaticum* (Fig. 1). Genetic identity between *C.lagenariiforme* and *C.angulosum* (CBS 140692), the closest species, shows 93.12% in *act* and 87.62% in *tef1*. *Cladosporiumlagenariiforme* and *C.angulosum* produce up to 6 and 14 conidia in long-branched chains, respectively, which is more than that in *C.xanthochromaticum* [up to 6(–7)] isolated from indoor environments (Bensch et al. 2018). The conidiophores of *C.lagenariiforme* are verrucose, and ramoconidia rarely have up to two septa, compared to that of the two sister species with smooth conidiophores and often aseptate ramoconidia (Sandoval-Denis et al. 2016). The ramoconidia of *C.lagenariiforme* are shorter than those of *C.angulosum* and *C.xanthochromaticum* (24.5–46 × 2–3.5 μm and 18–36 × 2–3.5 μm, respectively) (Sandoval-Denis et al. 2016).

#### ﻿ Cladosporium maltirimosum

Taxon classificationFungiCladosporialesCladosporiaceae

Wonjun Lee & Y.W. Lim sp. nov.

23D3A653-88B1-588E-AC60-58A90A813134

 847099

Figs 2B , 3B

##### Typification.

Republic of Korea. Jeollanam-do, Muan-gun, 35°03′44″N, 126°20′13″E, sea sand, Jul 2021, M.S. Park & Y.W. Lim (holotype SFC20230103-M51, stored in a metabolically inactive state).

##### Etymology.

The epithet ‘*maltirimosum*’ refers to the cracked colony that appears on the MEA medium derived from Latin rimosus (cracked).

##### Description.

**Asexual morphology: *Mycelium*** immersed, composed of septate, branched, hyaline to subhyaline, verruculose hyphae, nodulose, 2–5.5 μm wide. ***Conidiophores*** macronematous and micronematous, arising laterally from hyphae, septate, erect to slightly flexuous, non-nodulose, unbranched, up to 278 μm long, 1.9–4.3 μm wide, pale brown, verruculose. ***Conidiogenous cells*** integrated, terminal and intercalary, cylindrical to subcylindrical, 16–45.7 × 2.7– 4.1 μm, bearing up to three protuberant, slightly darkened, and refractive conidiogenous loci. ***Ramoconidia*** 0(−1)-septate, subcylindrical to ellipsoidal, 8.4–23 × 2.2–5.2 μm [av. (± SD)15.3 (± 4.5) × 3.6 (± 0.77)], pale brown, verruculose. ***Conidia*** solitary or forming branched chains, with up to seven conidia in the terminal unbranched part, aseptate, pale green, smooth to verruculose, with protuberant, slightly darkened, and refractive hila. ***Small terminal conidia*** aseptate, ellipsoidal, 4.2–6.7 × 2.2–3.3 μm [av. (± SD) 5.4 (± 0.61) × 2.8 (± 0.29)]. ***Intercalaryconidia*** aseptate, ellipsoidal to limoniform, rarely fusiform, 4.6–6.7 × 1.8–3.5 μm [av. (± SD) 5.5 (± 0.5) × 2.7 (± 0.35)]. ***Secondary ramoconidia*** 0(−1)-septate, subcylindrical to ellipsoidal, 8.9–22.5 × 2.7–5.6 μm [av. (± SD) 15.6 (± 3.34) × 3.6 (± 0.67)].

***Cultural characters***: Colonies on PDA 44–63 mm diam after 14 d at 25 °C, olive yellow (2D8) to olive (3E8), reverse dark gray (1F1), powdery, floccose, sometimes slightly fluffy, radially furrowed, wrinkled; margin white edge, slightly undulated or lobate, sometimes regular; aerial mycelia formed, moderate, without prominent exudates, sporulation profuse. Colonies on MEA 34–36 mm diam after 14 d at 25 °C, gray (1B1 to 1E1), reverse concolorous to dark gray (1F1), slightly crateriform due to cracked medium, floccose, woolly, radially furrowed, wrinkled; margin white, undulate; aerial mycelia abundantly formed, without prominent exudates, sporulation profuse. Colonies on OA 52–63 mm diam after 14 d at 25 °C, grayish green (1D3) to olive (1E5), reverse concolorous, powdery, floccose, fluffy, sometimes woolly, radially furrowed, wrinkled, flat; margin yellowish white (1A2), regular; aerial mycelia formed in spots, sometimes without prominent exudates, sporulation profuse. Colonies on SNA 37–39 mm diam after 14 d at 25 °C, grayish green (1D4) to olive (1E4), reverse concolorous to olive (3F8), powdery, flat; margin whitish, undulate; aerial mycelia absent, without prominent exudates, sporulation profuse.

##### Habitat and distribution.

Isolated from mudflats, sea sands, and seaweeds; Eastern, Southern, and Western Korean seaside in Republic of Korea.

##### Additional cultures examined.

Republic of Korea, Jeju-do, Hallim-eup, 33°23′53″N, 126°14′24″E, seaweed, Jul 2021, M.S. Park & Y.W. Lim (SFC20230103-M41, stored in a metabolically inactive state); Jeju-do, Chuja-myeon, 33°57′10″N, 126°20′18″E, seaweed, 31 Aug 2021, M.S. Park & Y.W. Lim (SFC20230103-M42, stored in a metabolically inactive state); Jeju-do, Chuja-myeon, 33°57′11.88″N, 126°18′07.56″E, seaweed, 31 Aug 2021, M.S. Park & Y.W. Lim (SFC20230103-M43, stored in a metabolically inactive state); Jeju-do, Hallim-eup, 33°23′53″N, 126°14′24″E, seaweed, 15 Aug 2021, M.S. Park & Y.W. Lim (SFC20230103-M44, stored in a metabolically inactive state); Jeollanam-do, Muan-gun, 35°01′40″N, 126°25′16″E, mudflat, Jan 2020, M.S. Park & Y.W. Lim (SFC20230103-M45, stored in a metabolically inactive state); Gangwon-do, Goseong-gun, 38°28′39″N, 128°26′22″E, Sea sand, 31 Oct 2016, M.S. Park & Y.W. Lim (SFC20170718-M12, stored in a metabolically inactive state); Ulsan, Ulju-gun, 35°23′00″N, 129°20′46″E, sea sand, Apr 2017, M.S. Park & Y.W. Lim (SFC20230103-M47, stored in a metabolically inactive state); Jeollanam-do, Suncheon-si, 34°50′29″N, 127°29′09″E, Sea sand, Jan 2020, M.S. Park & Y.W. Lim (SFC20230103-M48, stored in a metabolically inactive state); Gangwon-do, Goseong-gun, 38°28′39″N, 128°26′22″E, sea sand, Jan 2020, M.S. Park & Y.W. Lim (SFC20230103-M49, stored in a metabolically inactive state); Jeollanam-do, Suncheon-si, 34°50′29″N, 127°29′09″E, sea sand, Jul 2020, M.S. Park & Y.W. Lim (SFC20230103-M50, stored in a metabolically inactive state); Jeollanam-do, Suncheon-si, 34°50′29″N, 127°29′09″E, sea sand, Jul 2021, M.S. Park & Y.W. Lim (SFC20230103-M52, stored in a metabolically inactive state).

##### Notes.

*Cladosporiummaltirimosum* sp. nov. is closely related to *C.anthropophilum* and *C.devikae* and formed a well-supported clade (Fig. 1). The colonies of *C.maltirimosum* grow faster than those of *C.anthropophilum* associated with human (17–39 mm, 27–32 mm, and 23–26 mm, respectively) on PDA, MEA, and OA (Sandoval-Denis et al. 2016). However, whereas the growth rate of *C.maltirimosum* on PDA and OA falls within the range of the indoor *C.anthropophilum* colonies (17–80 mm and 27–74 mm, respectively), the colonies on MEA grow slower than the indoor *C.anthropophilum* (50–72 mm) (Bensch et al. 2018). *Cladosporiummaltirimosum* has wider hyphae (2–3 μm) and fewer maximum numbers of conidiogenous loci [3–8(–10)] than in *C.anthropophilum* (Sandoval-Denis et al. 2016). In addition, the ramoconidia of *C.maltirimosum* are significantly shorter than those of *C.anthropophilum* (20–42 × 2–5 μm; Sandoval-Denis et al. 2016). *Cladosporiummaltirimosum* produces up to six conidia, whereas *C.anthropophilum* produces up to four in an unbranched chain (Sandoval-Denis et al. 2016). The colonies of *C.maltirimosum* grow slower than those of *C.devikae* (70 mm diam) on PDA (Prasannath et al. 2021). Also, secondary ramoconidia of *C.maltirimosum* is longer than those of *C.devikae* (5–11 × 2–4 μm) (Prasannath et al. 2021), but the conidiophores are shorter than those of *C.devikae* (200–700 μm) (Prasannath et al. 2021). *Cladosporiummaltirimosum* showed a genetic identity of about 96% with *C.anthropophilum* (CBS 140685) (96.89% in *act* and 96.585% in *tef1*) but showed a high level of identity with *C.devikae* (BRIP 72278a) (100% in *act* and 97.56% in *tef1*). Despite having considerable genetic similarities with *C.devikae*, *C.maltirimosum* had distinct clades, different from *C.devikae*, in the phylogenetic tree. Furthermore, the two species can be distinguished by morphological differences such as growth rate on PDA and length of conidiophore and secondary ramoconidia. Aside from that, as *C.devikae* employs a single strain, there is not much information on the species, and the species’ phylogenetic placement within *Cladosporium* is unclear. Hence, additional study on *C.devikae* appears to be required.

#### ﻿ Cladosporium marinisedimentum

Taxon classificationFungiCladosporialesCladosporiaceae

Wonjun Lee & Y.W. Lim sp. nov.

9DDAE5ED-3C17-5BEE-8119-571B369B13F1

 847100

Figs 2C , 3C

##### Typification.

Western Pacific Ocean, 15°22.697'N, 151°40.836'E, depth 5730 m, deep-sea sediment, 23 May 2021, Y.J. Kim, Wonjun Lee & Y.W. Lim (holotype SFC20230103-M28, stored in a metabolically inactive state).

##### Etymology.

The epithet ‘*marinisedimentum*’, derived from Latin, refers to ‘marine sediment,’ a habitat where the species was isolated.

##### Description.

**Asexual morphology: *Mycelium*** immersed, composed of septate, branched, pale brown or subhyaline, verruculose hyphae, 2.6–4.5 μm wide. ***Conidiophores*** macronematous and micronematous, arising laterally or terminally from hyphae, sometimes reduced to conidiogenous cells, septate, erect to slightly flexuous, non-nodulose, branched, up to 220 μm long, 1.5–3.7 μm wide, pale brown, smooth to verruculose. ***Conidiogenous cells*** integrated, terminal, rarely intercalary, filiform to cylindrical, 16.2–41.5 × 2–3.2 μm, bearing up to three slightly darkened and refractive conidiogenous loci. ***Ramoconidia*** 0–1(–3)-septate, subcylindrical to cylindrical, 9.2–31.8 × 1.7–3.3 μm [av. (± SD) 17.4 (± 6.07) × 2.6 (± 0.34)], pale brown, smooth to verruculose. ***Conidia*** forming branched chains, with up to four conidia in the terminal unbranched part, aseptate, pale brown, smooth to verruculose, with protuberant, slightly darkened, and refractive hila. ***Small terminal conidia*** aseptate, subglobose to ellipsoidal, 2.6–4.6 × 2.1–3.2 μm [av. (± SD) 3.6 (± 0.52) × 2.6 (± 0.23)]. ***Intercalaryconidia*** 0(−1)-septate, subglobose to ellipsoidal-limoniform, ovoid, 3.2–9.1 × 2.1–3.6 μm [av. (± SD) 4.6 (± 1.25) × 2.8 (± 0.34)]. ***Secondary ramoconidia*** 0–1(−2)-septate, subcylindrical to ellipsoidal, 7.8–31.6 × 2.1–3.4 μm [av. (± SD) 13.9 (± 4.88) × 2.7 (± 0.34)].

***Cultural characters***: Colonies on PDA 37–46 mm diam after 14 d at 25 °C, olive (2E6 to 3E8), reverse dark gray (1F1), velvety, powdery, radially furrowed, wrinkled, umbonate; margin white edge, slightly lobate; aerial mycelia sparsely formed, without prominent exudates, sporulation profuse. Colonies on MEA 36–43 mm diam after 14 d at 25 °C, olive gray (2D2) to olive (2E3), reverse dark gray (1F1), powdery, velvety, undulate, slightly raised, radially furrowed, wrinkled; margin whitish edge, slightly lobate or undulate; aerial mycelia moderately formed, somewhat irregular, without prominent exudates, sporulation profuse. Colonies on OA 33–37 mm diam after 14 d at 25 °C, olive gray (2E2) to olive (2F3), reverse concolorous, powdery, floccose, flat; margin whitish, regular; aerial mycelia abundantly formed in radial form, without prominent exudates, sporulation profuse. Colonies on SNA 19–23 mm diam after 14 d at 25 °C, olive (2F3 to 2F8), reverse concolorous, powdery, flat; margin yellowish white (1A2), regular; aerial mycelia sparsely formed, without prominent exudates, sporulation profuse.

##### Habitat and distribution.

Isolated from deep-sea sediments and sea sands; Eastern and Southern Korean seaside in Republic of Korea and Eastern Mariana trench in Western Pacific Ocean.

##### Additional cultures examined.

Western Pacific Ocean, 16°06′15″N, 152°25′00″E, depth 5814 m, deep-sea sediment, 27 May 2021, Y.J. Kim, Wonjun Lee & Y.W. Lim (MABIK FU00001143, stored in a metabolically inactive state); Republic of Korea. Gangwon-do, Goseong-gun, 38°28′39″N, 128°26′22″E, sea sand, Jul 2021, M.S. Park & Y.W. Lim (SFC20230103-M29, stored in a metabolically inactive state).

##### Notes.

*Cladosporiummarinisedimentum* sp. nov. is phylogenetically related to *C.sphaerospermum*. The former species has broader hyphae than the latter one (1–3 μm) (Bensch et al. 2018), but the conidiophores of *C.marinisedimentum* are narrower than that of *C.sphaerospermum* (2.5–4.5(–6) μm) (Bensch et al. 2018). The number of septa in ramoconidia and secondary ramoconidia in *C.marinisedimentum* is lower than that in *C.sphaerospermum* (up to five septa and 0–3(–4)-septate; Bensch et al. 2018). On PDA, *C.marinisedimentum* does not produce any exudates or pigments, whereas *C.sphaerospermum* produces prominent exudates and green soluble pigments (Bensch et al. 2018). Furthermore, the two species differ from each other in the identities of *act* (98.04%) and *tef1* (93.23%) genetic markers (CBS 193.54).

#### ﻿ Cladosporium marinum

Taxon classificationFungiCladosporialesCladosporiaceae

Wonjun Lee & Y.W. Lim sp. nov.

DFB9BB50-6EB4-5C1D-840D-BB8BA8A8378B

 847098

Figs 2D , 3D

##### Typification.

Republic of Korea. Jeju-do, Chuja-myeon, 33°23′53″N, 126°14′24″E, seaweed, 31 Aug 2021, M.S. Park & Y.W. Lim (holotype SFC20230103-M33, stored in a metabolically inactive state).

##### Etymology.

The name ‘*marinum*’, derived from Latin, refers to the various marine substrates (seaweed, sea sand, and mudflat) from which the species was isolated.

##### Description.

**Asexual morphology: *Mycelium*** superficial and immersed, composed of septate, branched, hyaline to subhyaline, smooth to verruculose hyphae, nodulose, 2–5.7 μm wide. ***Conidiophores*** macronematous and micronematous, arising laterally or terminally from hyphae, (sometimes reduced to conidiogenous cells), septate, slightly flexuous, non-nodulose, usually unbranched or branched, up to 243 μm long, 2–4 μm wide, pale brown, verruculose to verrucose. ***Conidiogenous cells*** integrated, terminal and intercalary, cylindrical to subcylindrical, 20.5–47.7 × 2–3.6 μm, bearing up to four slightly darkened and refractive conidiogenous loci. ***Ramoconidia*** 0–1(–2)-septate, subcylindrical to cylindrical, 10.2–28.171 × 2.4–4.1 μm [av. (± SD) 17.3 (± 2.39) × 2.9 (± 0.37)], pale brown, verruculose. ***Conidia*** forming branched chains, with up to five conidia in the terminal unbranched part, long neck area between conidia, aseptate, pale brown, smooth to verruculose, with protuberant, slightly darkened and refractive hila. ***Small terminal conidia*** aseptate, obovoidal to ellipsoidal, 2.9–5.3 × 2–3 μm [av. (± SD) 3.8 (± 0.53) × 2.5 (± 0.25)]. ***Intercalaryconidia*** aseptate, very rarely 1-septate, ellipsoidal to limoniform, obovoid, sometimes subcylindrical, 2.7–6.2 × 1.8–2.8 μm [av. (± SD) 4.2 (± 0.76) × 2.3 (± 0.22)]. ***Secondary ramoconidia*** 0–1-septate, subcylindrical to cylindrical, slightly obclavate, 8.5–28.2 × 2.3–3.7 μm [av. (± SD) 14.5 (± 4.38) × 2.9 (± 0.35)].

***Cultural characters***: Colonies on PDA 37–66 mm diam after 14 d at 25 °C, olive (3E3 to 3F4), reverse dark gray (1F1), floccose-velvety, sometimes woolly, raised, radially furrowed, wrinkled, raised; margin white, slightly hyaline edge, undulated; aerial mycelia abundantly formed, with numerous prominent exudates, sporulation profuse. Colonies on MEA 35–40 mm diam after 14 d at 25 °C, grayish white (1B1) to olive gray (3D2), reverse dark gray (1F1), velvety, raised to crateriform, radially furrowed at the margin region, wrinkled; margin white, regular; aerial mycelia abundantly formed, dense, without prominent exudates, sporulation profuse. Colonies on OA 41–47 mm diam after 14 d at 25 °C, gray (1C1 to 1E1), reverse concolorous, velvety, floccose, raised, radially furrowed; margin yellowish, regular; aerial mycelia abundantly formed, with few exudates, sporulation profuse. Colonies on SNA 37–41 mm diam after 14 d at 25 °C, olive (3D3) to olive yellow (3E8), reverse olive (3D3 to 3F3), slightly fluffy-floccose, powdery, flat; margin yellowish white (2A2), hyaline, undulate; aerial mycelia abundantly moderate, without prominent exudates, sporulation profuse.

##### Habitat and distribution.

Isolated from mudflats, sea sands, and seaweeds; Southern and Western Korean seaside in Republic of Korea.

##### Additional cultures examined.

Republic of Korea. Jeju-do, Chuja-myeon, 33°23′53″N, 126°14′24″E, seaweed, 31 Aug 2021, M.S. Park & Y.W. Lim (SFC20230103-M30, stored in a metabolically inactive state); Jeju-do, Chuja-myeon, 33°23′53″N, 126°14′24″E, seaweed, 31 Aug 2021, M.S. Park & Y.W. Lim (SFC20230103-M31, stored in a metabolically inactive state); Jeju-do, Chuja-myeon, 33°23′53″N, 126°14′24″E, seaweed, 31 Aug 2021, M.S. Park & Y.W. Lim (SFC20230103-M32, stored in a metabolically inactive state); Jeju-do, Chuja-myeon, 33°57′50″N, 126°17′38″E, seaweed, 31 Aug 2021, M.S. Park & Y.W. Lim (SFC20230103-M34, stored in a metabolically inactive state); Jeju-do, Hallim-eup, 33°23′53″N, 126°14′24″E, seaweed, 15 Aug 2021, M.S. Park & Y.W. Lim (SFC20230103-M35, stored in a metabolically inactive state); Jeju-do, Hallim-eup, 33°23′53″N, 126°14′24″E, seaweed, 15 Aug 2021, M.S. Park & Y.W. Lim (SFC20230103-M36, stored in a metabolically inactive state); Jeollanam-do, Muan-gun, 35°01′40″N, 126°25′16″E, mudflat, Apr 2017, M.S. Park & Y.W. Lim (SFC20230103-M37, stored in a metabolically inactive state); Jeollanam-do, Muan-gun, 35°03′44″N, 126°20′13″E, sea sand, Oct 2016, M.S. Park & Y.W. Lim (SFC20230103-M38, stored in a metabolically inactive state); Jeollanam-do, Suncheon-si, 34°50′29″N, 127°29′09″E, sea sand, Jul 2020, M.S. Park & Y.W. Lim (SFC20230103-M39, stored in a metabolically inactive state); Jeju-do, Seogwipo-si, 33°13′58″N, 126°18′47″E, sea sand, Jan 2021, M.S. Park & Y.W. Lim (SFC20230103-M40, stored in a metabolically inactive state).

##### Notes.

*Cladosporiummarinum* sp. nov. forms an independent clade in the phylogenetic tree of the *C.cladosporioides* species complex (Fig. 1), but it has no distinguishable characteristics compared to those of the other species. Molecular and phylogenetic approaches are imperative to identify and differentiate this species from other species.

#### ﻿ Cladosporium proteacearum

Taxon classificationFungiCladosporialesCladosporiaceae

Prasannath, Akinsanmi & R.G. Shivas

FA727F83-2FE1-51FB-8997-8D74E0C931DD

 841221

Figs 2E , 3E

##### Holotype.

BRIP 72301a (Prasannath et al. 2021).

##### Description.

**Asexual morphology: *Mycelium*** immersed, composed of septate, branched, pale brown, smooth to verruculose hyphae, 2–5 μm wide. ***Conidiophores*** macronematous and micronematous, arising laterally from hyphae, septate, erect to slightly flexuous, unbranched, up to 216 μm long, 2–4.5 μm wide, pale brown, verruculose. ***Conidiogenous cells*** integrated, terminal, cylindrical to subcylindrical, 18–59 × 2–4.5 μm, bearing up to three conidiogenous loci, slightly darkened and refractive. ***Ramoconidia*** 0–1(−2)-septate, subcylindrical to cylindrical, 9.2–38 × 2.3–4.7 μm [av. (± SD)24.1 (± 7.87) × 3.3 (± 0.58)], pale brown, smooth to verruculose. ***Conidia*** forming branched chains, with up to eight conidia in the terminal unbranched part, septate, pale brown, smooth to verruculose, slightly darkened and refractive hila. ***Small terminal conidia*** aseptate, ellipsoidal, 3.2–4.8 × 1.9–2.7 μm [av. (± SD) 3.9 (± 0.34) × 2.4 (± 0.2)]. ***Intercalaryconidia*** 0(–1)-septate, ellipsoidal, oblong-ellipsoidal, 3.4–7.6 × 2.1–3.6 μm [av. (± SD) 4.8 (± 0.83) × 2.5 (± 0.32)]. ***Secondary ramoconidia*** 0–1-septate, oblong-ellipsoidal, ellipsoidal, subcylindrical, 4.5–25.6 × 1.9–4.5 μm [av. (± SD) 10.2 (± 4.3) × 3 (± 0.5)].

***Cultural characters***: Colonies on PDA 28–44 mm diam after 14 d at 25 °C, olive (2F7) to grayish yellow (2B4), reverse dark gray (1F1), velvety, raised, radially furrowed, wrinkled; margin white edge, undulated or lobate; aerial mycelia sparse, without prominent exudates, sporulation profuse, crack formed. Colonies on MEA 20–40 mm diam after 14 d at 25 °C, grayish green (29D7), reverse olive brown (4F8), velvety, powdery, raised, radially furrowed, umbonate, wrinkled; margin white, slightly undulate; aerial mycelia abundantly formed in protruding area (in the center), regular, without prominent exudates, sporulation profuse. Colonies on OA 39–46 mm diam after 14 d at 25 °C, wax white (2B3) to olive (2E5), reverse concolorous, floccose, flat, sometimes wrinkled; margin yellowish white (1A2), regular; aerial mycelia formed in spot, without prominent exudates, sporulation profuse. Colonies on SNA 40–41 mm diam after 14 d at 25 °C, olive yellow (2C7) to olive (1E4), reverse olive (2F5), powdery, flat; margin hyaline, regular; aerial mycelia sparse, without prominent exudates, sporulation profuse.

##### Habitat and distribution.

Isolated from plant material (Racames of *Macadamiaintegrifolia*), sea sands, and seaweeds; Australia and Eastern, Southern, and Western Korean seaside and in Republic of Korea.

##### Additional cultures examined.

Republic of Korea. Ulsan, Ulju-gun, 35°23′00″N, 129°20′46″E, sea sand, Jan 2021, M.S. Park & Y.W. Lim (SFC20230103-M55, stored in a metabolically inactive state); Jeju-do, Hallim-eup, 33°23′53″N, 126°14′24″E, seaweed, Jul 2021, M.S. Park & Y.W. Lim (SFC20230103-M53, stored in a metabolically inactive state); Incheon, Ganghwa-gun, 37°35′18″N, 126°26′33″E, sea sand, Jan 2021, M.S. Park & Y.W. Lim (SFC20230103-M54, stored in a metabolically inactive state).

##### Notes.

This strain grows slower and forms less flat colonies on PDA compared to the holotype colonies (70 mm diam, flat) of *C.proteacearum* (Prasannath et al. 2021). The characteristics of this species on MEA, OA, and SNA media were unavailable because the description on these media is insufficient (Prasannath et al. 2021). Septa are visible in the secondary ramoconidia and intercalary conidia of this strain, but they have not been reported for the holotype (Prasannath et al. 2021). To our knowledge, this is the first report of this species from the Republic of Korea and from marine environments.

#### ﻿ Cladosporium snafimbriatum

Taxon classificationFungiCladosporialesCladosporiaceae

Wonjun Lee & Y.W. Lim sp. nov.

D8C94BFF-2F21-5265-AC06-9CDA49E40839

 847093

Figs 2F , 3F

##### Typification.

Republic of Korea, Incheon, Ganghwa-gun, 37°36′37″N, 126°31′24″E, mudflat, 12 Jun 2019, M.S. Park & Y.W. Lim (holotype: SFC20230103-M65, stored in a metabolically inactive state).

##### Etymology.

The epithet ‘*snafimbriatum*’, derived from Latin, refers to the fimbriate margin that appears on the SNA medium.

##### Description.

**Asexual morphology: *Mycelium*** immersed, composed of septate, branched, hyaline to subhyaline, verruculose hyphae, nodulose, 3–7.8 μm wide. ***Conidiophores*** macronematous and micronematous, arising laterally or terminally from hyphae, sometimes reduced to conidiogenous cells, septate, solitary, erect or somewhat flexuous, non-nodulose, sometimes slightly geniculate, usually unbranched, up to 294 μm long, 2.7–4.2 μm wide, pale brown, verruculose to verrucose. ***Conidiogenous cells*** integrated, terminal or intercalary, cylindrical, 9.6–48 × 2.1–4.8 μm, bearing up to four slightly darkened and refractive conidiogenous loci. ***Ramoconidia*** 0–2(−3)-septate, subcylindrical, somewhat slightly clavate, 13.3–39.7 × 2.7–4.5 μm [av. (± SD) 22.5 (± 6.32) × 3.5 (± 0.43)], pale brown, verruculose to verrucose. ***Conidia*** solitary or forming branched chains, with up to five conidia in the terminal unbranched part, sometimes long and thickened neck between conidia, 0(−1) septate, pale brown, verruculose to verrucose, with protuberant, slightly darkened, and refractive hila. ***Small terminal conidia*** 0(−1) septate, ellipsoidal to subcylindrical, clavate, 4.7–8.3 × 2.9–4.5 μm [av. (± SD) 6.1 (± 0.81) × 3.5 (± 0.35)]. ***Intercalaryconidia*** 0–1(−2) septate, ellipsoidal to subcylindrical, limoniform, clavate, sometimes obclavate, 6.5–14.4 × 2.7–3.9 μm [av. (± SD) 8.5 (± 1.82) × 3.3 (± 0.28)]. ***Secondary ramoconidia*** 0–2(−3)-septate, subcylindrical to cylindrical, 12.5–29.9 × 2.9–4.2 μm [av. (± SD) 18.8 (± 3.86) × 3.5 (± 0.34)].

***Cultural characters***: Colonies on PDA 47–52 mm diam after 14 d at 25 °C, grayish green (30B3) to deep green (30E3), reverse dark gray (1F1), umbonate with slightly elevated central colony, velvety, radially furrowed, wrinkled at the center, sometimes frequently wrinkled on the entire colony; margin undulated, white edge; aerial mycelia moderate, without prominent exudates, sporulation profuse. Colonies on MEA 40–48 mm diam after 14 d at 25 °C, greenish gray (29B2) to grayish green (30C3), reverse dull green (30D3) to dark green (30F3), slightly raised colony, velvety; margin undulated, white edge, sometimes hyaline, radially furrowed, wrinkled; aerial mycelia abundantly formed, dense, somewhat irregular, without prominent exudates, sporulation profuse. Colonies on OA 35–38 mm diam after 14 d at 25 °C, deep green (30E8), reverse concolorous, powdery, slightly floccose, flat; margin yellowish, regular; aerial mycelia sparsely formed in the center, regular, without prominent exudates, sporulation profuse. Colonies on SNA 25–30 mm diam after 14 d at 25 °C, deep green (30E8) to dark green (30F8), reverse concolorous to dark gray (1F8), powdery, slightly floccose in the center, flat; margin olive (2E8 to 2F8), fimbriate; aerial mycelia sparsely to dense, irregular, without prominent exudates, sporulation profuse.

##### Habitat and distribution.

Isolated from mudflats; Western Korean seaside in Republic of Korea.

##### Additional cultures examined.

Republic of Korea, Incheon, Ganghwa-gun, 37°36′36″N, 126°31′13″E, mudflat, 12 Jun 2019, M.S. Park & Y.W. Lim (SFC20230103-M63, stored in a metabolically inactive state); Incheon, Ganghwa-gun, 37°36′36″N, 126°31′13″E, mudflat, 12 Jun 2019, M.S. Park & Y.W. Lim (SFC20230103-M64, stored in a metabolically inactive state).

##### Notes.

This species has a distinguishing characteristic of forming a fimbriate margin on the SNA medium. *Cladosporiumsnafimbriatum* is phylogenetically distant with identities of 94.87% for *act* and 90.73% for *tef1*, compared to *C.allicinum* (CBS 121624) (Bensch et al. 2012). *Cladosporiumsnafimbriatum* and *C.allicinum* (Bensch et al. 2012) belong to the same clade and their conidial structures are similar (Bensch et al. 2012). However, the conidiophores of *C.snafimbriatum* are rougher than those of *C.allicinum*, and small terminal conidia and intercalary conidia are septate (aseptate in *C.allicinum*) (Bensch et al. 2012). *Cladosporiumsnafimbriatum* grows faster on PDA (22–32 mm), MEA (21–32 mm), and OA (20–32 mm) than *C.allicinum* (Bensch et al. 2012).

### ﻿Diversity

The *Cladosporium* strains were isolated from three types of marine habitats, namely seawater, seaweed, and sediment, from two districts (Table 2). In the open sea in the Western Pacific, two species were detected in seawater (*C.halotolerans*) and sediments (*C.halotolerans* and *C.marinisedimentum* sp. nov.). *Cladosporiumhalotolerans* was detected from two districts and all three marine habitats (Table 2). In the intertidal zone of the Republic of Korea, 14 species were discovered from seaweeds (eight species) and sediments (13 species). *Cladosporiumlagenariiforme* sp. nov. was isolated only from seaweed. Six species (*C.allicinum*, *C.marinisedimentum* sp. nov., *C.snafimbriatum* sp. nov., *C.velox*, *C.xanthochromaticum*, and *C.xylophilum*) were only found in the sediment.

**Table 2.** Sites and habitats where *Cladosporium* species were isolated. In the site column, Western Korea, Southern Korea, Eastern Korea, and Western Pacific are represented by the acronym WK, SK, EK, and WP, respectively. “o” indicates that the species was isolated at the corresponding site or the habitat. The new species are in bold.

Species complex	Species	Site				Habitat		
		WK	SK	EK	WP	Seaweed	Sediment	Seawater
*C.cladosporioides*	***C.lagenariiforme***		**o**			**o**		
	***C.maltirimosum***	**o**	**o**	**o**		**o**	**o**	
	***C.marinum***	**o**	**o**			**o**	**o**	
	*C.perangustum*	o	o			o	o	
	*C.proteacearum*	o	o	o		o	o	
	*C.rectoides*	o	o	o		o	o	
	*C.tenuissimum*	o	o			o	o	
	*C.xanthochromaticum*	o					o	
	*C.xylophilum*	o		o			o	
*C.herbarum*	*C.allicinum*		o				o	
	***C.snafimbriatum***	**o**					**o**	
*C.sphaerospermum*	*C.halotolerans*	o	o	o	o	o	o	o
	***C.maresedimentum***			**o**	**o**		**o**	
	*C.velox*	o					o	

## ﻿Discussion

*Cladosporium* is a cosmopolitan genus, and several of its species are found in various marine environments. We isolated *Cladosporium* strains from three different habitats in the intertidal zone in the Republic of Korea and in the open sea in the Western Pacific. Fourteen *Cladosporium* species (88 strains) were identified based on the analyses of ITS, *act*, and *tef1* marker sequences. Five species (*C.lagenariiforme*, *C.maltirimosum*, *C.marinisedimentum*, *C.marinum*, and *C.snafimbriatum*) are being reported for the first time. The diversity of *Cladosporium* in marine environments is much higher than previously reported (Jones et al. 2019).

The topology of the phylogenetic tree generated for *Cladosporiums. str.* was analogous to that for *Cladosporium* in previous taxonomic studies (Sandoval-Denis et al. 2016; Ma et al. 2017) (Fig. 1). Nine species (*C.allicinum*, *C.halotolerans*, *C.perangustum*, *C.proteacearum*, *C.rectoides*, *C.tenuissimum*, *C.velox*, *C.xanthochromaticum*, and *C.xylophilum*) matched the reference sequences of known species in the phylogenetic trees (Fig. 1). *Cladosporiumproteacearum* was isolated from the marine environments (seaweed and marine sediments) for the first time in this study, whereas others have already been reported (Garzoli et al. 2018; Liu et al. 2020; Luo et al. 2020; Borzykh et al. 2022). Phylogenetic analyses and morphological characteristics support the notion that the remaining five species are new to science. These five species (*C.lagenariiforme*, *C.maltirimosum*, *C.marinisedimentum*, *C.marinum*, and *C.snafimbriatum*) formed each distinct clades as new species to be distinguished with sister species (Fig. 1). Also, distinct morphological differences between the new species and their closely related species are described in the Notes in the Taxonomy section. These morphological characteristics of *Cladosporium* have been described when proposing new species using molecular data (Bensch et al. 2012, 2018; Pereira et al. 2022).

The environmental conditions in the two districts (the intertidal zone in the Republic of Korea and the open sea in the Western Pacific) are distinct. The Western Pacific deep sea, where *C.halotolerans* and *C.marinisedimentum* sp. nov. were isolated (Table 2), is an environment under high pressure and cold temperature, which hinders the activation and growth of fungi. However, *Cladosporium* can adapt to harsh marine conditions by possessing halotolerant and psychrotolerant properties. *Cladosporium* has been reported to grow under 40 MPa (= 400 bar) (Peng et al. 2021) and in cold conditions (Dhakar and Pandey 2016), and continuously isolated from deep-sea environments (Singh et al. 2012; Luo et al. 2020; Marchese et al. 2021). Thus, *Cladosporium* species are presumed to survive as dormant spores under harsh conditions. *Cladosporiumhalotolerans* is the only *Cladosporium* species isolated from seawater habitats in the Western Pacific in this study. Seawater is a low-density habitat that diffuses quickly. To circumvent this, seawater was filtered to isolate marine fungi, but the quantity of seawater or sampling time may be insufficient. It is necessary to perform research on growing or germinating spores in the two environments to understand the role of *Cladosporium* in deep-sea environments.

In contrast, the intertidal zones are relatively warmer and under lower pressures. Various *Cladosporium* species inhabit seaweeds and sediments in the intertidal zones. Eight species (*C.halotolerans*, *C.lagenariiforme* sp. nov., *C.maltirimosum* sp. nov., *C.marinum* sp. nov., *C.perangustum*, *C.proteacearum*, *C.rectoides*, and *C.tenuissimum*) were isolated from seaweeds in this study, and many studies have verified that *Cladosporium* degrades seaweeds (Lee et al. 2019; Patyshakuliyeva et al. 2020). Seaweeds can accumulate in sediments and become nutrients for saprobes, such as *Cladosporium*. Recently, plastic waste has been flowing into the marine environments, and *Cladosporium* species isolated from such environments have been shown to possess the ability to degrade plastics (Kim et al. 2022; Zhang et al. 2022).

In sediments in the intertidal zone, where we found all species except *C.lagenariiforme* sp. nov., we found the most diversified *Cladosporium* species (*C.allicinum*, *C.halotolerans*, *C.maltirimosum* sp. nov., *C.marinisedimentum* sp. nov., *C.marinum* sp. nov., *C.perangustum*, *C.proteacearum*, *C.rectoides*, *C.snafimbriatum* sp. nov., *C.tenuissimum*, *C.velox*, *C.xanthochromaticum*, and *C.xylophilum*). Sediments in intertidal zone are rich in various nutrients through inputs from terrestrial environments and open sea, which may provide abundant nutrients for fungal survival or growth (Jickells 1998; Santos et al. 2021). Besides, the melanin production of *Cladosporium* may facilitate survival in the sediments affected by environmental stresses such as UV, salinity, and temporary dryness (Gunde-Cimerman et al. 2000; Gessler et al. 2014).

The diversity of *Cladosporium* species needs to be studied because *Cladosporium* has frequently been detected in marine environments (Suryanarayanan 2012; Jones et al. 2019; Luo et al. 2020). This work, which detected a total of fourteen *Cladosporium* species (including five new species), can broaden the understanding of the biodiversity and ecology of marine *Cladosporium* species. However, studies on mechanisms of fungal stress adaptation in extreme marine environment are scarce. In future, we will investigate the mechanisms of ecological adaptation of *Cladosporium* species in marine environments and intend to study the different secondary metabolites produced by them for adapting to a wide range of environments.

## Supplementary Material

XML Treatment for Cladosporium lagenariiforme

XML Treatment for Cladosporium maltirimosum

XML Treatment for Cladosporium marinisedimentum

XML Treatment for Cladosporium marinum

XML Treatment for Cladosporium proteacearum

XML Treatment for Cladosporium snafimbriatum

## ﻿Additional information

### Author ORCIDs

Wonjun Lee https://orcid.org/0000-0002-7227-0777

Ji Seon Kim https://orcid.org/0000-0003-1869-7347

Chang Wan Seo https://orcid.org/0000-0002-5948-1836

Jun Won Lee https://orcid.org/0000-0003-1416-3428

Sung Hyun Kim https://orcid.org/0000-0002-5505-9451

Yoonhee Cho https://orcid.org/0000-0002-0743-0914

Young Woon Lim https://orcid.org/0000-0003-2864-3449

### Data availability

All of the data that support the findings of this study are available in the main text or Supplementary Information.

## Supplementary materials

Supplementary material 1 Strains of *Cladosporium* isolated in this study with detailed information on habitats and regions and GenBank accession numbers Data type tables (Excel file) https://binary.pensoft.net/file/858785This dataset is made available under the Open Database License (http://opendatacommons.org/licenses/odbl/1.0/). The Open Database License (ODbL) is a license agreement intended to allow users to freely share, modify, and use this Dataset while maintaining this same freedom for others, provided that the original source and author(s) are credited.Wonjun Lee, Ji Seon Kim, Chang Wan Seo, Jun Won Lee, Sung Hyun Kim, Yoonhee Cho, Young Woon Lim
